# A Precision Computational Framework for sLORETA Neurofeedback in Mild Cognitive Impairment: Integration of qEEG Biomarkers and Neuropsychological Metrics

**DOI:** 10.3390/ijerph23050624

**Published:** 2026-05-08

**Authors:** Viviane Dasilva, Diana Poli, Olimpia Pino

**Affiliations:** 1 Department of Medicine and Surgery, University of Parma, 43126 Parma, Italy; vivianedasilva@brain-performance.eu (V.D.); olimpia.pino@unipr.it (O.P.); 2 Department of Occupational and Environmental Medicine, Epidemiology and Hygiene, INAIL (The National Institute for Insurance Against Accidents at Work), 00040 Monte Porzio Catone, Italy

**Keywords:** neurofeedback, mild cognitive impairment, computational neuroscience, individual Alpha frequency, sLORETA, aperiodic 1/f, Bayesian adaptive control, Clinical Deviation Index

## Abstract

**Highlights:**

**Public health relevance—How does this work relate to a public health issue?**

- Mild Cognitive Impairment (MCI) represents a critical global health challenge, serving as a vital window for preventive intervention before progression toward irreversible dementia.
- This article addresses the urgent need for non-invasive Personalized Digital Therapeutics (DTx) to mitigate the socio-economic burden associated with an increasingly aging global population.

**Public health significance—Why is this work of significance to public health?**

- This paper introduces a novel mathematical framework that integrates qEEG biomarkers, Individual Alpha Peak Frequency (i-APF), and the Clinical Deviation Index (CDI) with systems biology, providing superior diagnostic and rehabilitative precision compared to standard fixed-band protocols.
- The proposed approach shifts cognitive deficit treatment from symptomatic management to neurophysiological recalibration based on neural attractor stability and Long-Term Potentiation (LTP).

**Public health implications—What are the key implications or messages for practitioners, policy makers and/or researchers in public health?**

- For clinicians and researchers, the model establishes the necessity of accounting for aperiodic noise (1/f) and the Weber Threshold to personalize operant reinforcement and enhance sLORETA neurofeedback efficacy.
- For policy makers, this paper advocates for the adoption of Digital Twin models and Bayesian DWS (BDWS) algorithms as accessible precision medicine standards capable of inducing retroactive reversion of cognitive symptoms.

**Abstract:**

This paper proposes a high-precision theoretical and computational neurorehabilitation framework for Mild Cognitive Impairment (MCI), connecting computational neuroscience and clinical practice through qEEG-guided neurofeedback training (NFT). By employing sLORETA to identify putative pathological nodes within the Default Mode Network (DMN)—specifically the Precuneus and the Posterior Cingulate—the model utilizes spectral decomposition to isolate the aperiodic 1/f component, reducing background noise bias and allowing the calculation of a pure individual alpha frequency (IAF) to inform recalibration of Weber’s Cognitive Threshold. The core architecture uses Bayesian algorithms and stochastic modeling to drive a Dynamic Weight Change mechanism. To support Long-Term Potentiation (LTP) and Hebbian learning, reward thresholds are modulated in real time to target a 70% success rate, as a strategic rationale to anticipate neural fatigue while maintaining the Reward Prediction Error required for synaptic strengthening. As a prospective validation pathway, future studies may assess clinical value through changes in MoCA and RAVLT scores, as well as by examining normalization of cortical coherence in the Default Mode Network (DMN). By merging computational neuroscience with biological models of synaptic plasticity, this work outlines how individual biology can be mapped into an explicit mathematical model. The proposed framework may inform an individualized protocol that provides an objective model-based measure of cognitive recovery, suggesting a replicable and robust strategy for neurorehabilitation during the prodromal phase of dementia, and providing a new approach to neuroscience-based cognitive rehabilitation. This work is intended as a theoretical and computational framework; no complete empirical dataset is reported in the present manuscript.

## 1. Introduction

For over 90 years, electroencephalography (EEG) has served as a primary object of investigation within both rigorous research environments and specialized clinical practices [1]. In recent years, bolstered by significant technological advances, the role of EEG has undergone a profound transformation, evolving from a traditional diagnostic support tool into a dynamic instrument for active neurological rehabilitation [2,3]. This progress toward precision cognitive rehabilitation is currently guided by the integration of high-resolution quantitative EEG (qEEG) systems and sophisticated computational neuroscience models, which utilize complex mathematical frameworks to achieve unprecedented precision in target calculations and therapeutic delivery [4]. This paradigm shift is further supported by Brain–Computer Interface (BCI) technology, which enables seamless, real-time dialog between neural output and external modulation, creating a closed-loop system of recovery [5].

### 1.1. Background: From qEEG to Precision Neurofeedback

Neurofeedback, unlike pharmacological approaches or physically invasive interventions, operates through real-time perceptual stimulations based on both the continuous measurement of the characteristics of EEG signals and training in awareness and voluntary modulation of mental states [6]. From a conceptual point of view, the cardinal principle is the interaction between the external representation of the brain signal and the subject’s adaptive ability to modify it in response to stimuli and reinforcements. Typically, a qEEG recording acquired through standardized procedures (such as the international system 10–20) is broken down into frequency bands, Delta, Theta, Alpha1, Alpha 2, Beta-1, Beta-2, and Gamma, each associated with a specific neurocognitive condition [7]. For example, Theta and Delta are commonly related to states of low cortical activation or somnolence; Alpha is often associated with vigilant relaxation; Beta, in various sub-bands, is linked to concentration and focused cognitive activity; and Gamma is related to high cognitive functions. Modulation in Theta activity is correlated with cognitive functions, such as memory, attention, LTP, and LTD [8]. In MCI rehabilitation, neurofeedback training aims to amplify or selectively suppress these bands based on the pathological profile highlighted by the qEEG. Initial calibration requires the determination of the “target” electrical state through quantitative analyses such as Z-score mapping. In this way, statistically significant deviations from the norm can be defined.

A cornerstone of modern qEEG is the implementation of sLORETA (Standardized Low-Resolution Brain Electromagnetic Tomography). This method enables the localization of cortical electrical activity within the primary nodes of core Intrinsic Connectivity Networks (ICNs). These networks include the Default Mode Network (DMN), the Central Executive Network (CEN), and the Salience Network (SN). By isolating these nodes, sLORETA offers a high-fidelity, dynamic picture of the brain’s functional architecture, allowing for the precise targeting of deep-seated cortical structures [9,10,11].

Three-dimensional localized abnormalities with sLORETA-type techniques provide a spatial reference to identify cortical areas involved in feedback [8]. For example, if the qEEG profile shows Theta hyper-coherence in posterior temporal regions or frontal slowdowns of use, session algorithms can configure tasks and visual incentives associated with its reduction [7]. Operating modes include responsive audio-visual systems: video sequences or animations that change state depending on the desired direction of EEG activity. The temporal continuity of the feedback is essential to allow the user to directly associate their internal state with the perceived external change. According to Peterson et al. [7], protocols can target training mainly on one hemisphere while maintaining attention to the interconnected global network, under the hypothesis that local modification also induces functional reorganization at the systemic level. However, some placebo-controlled studies have raised questions about the specificity of the observed effect [12], suggesting the need for rigorous methodological criteria and large samples. In the context of MCI, the approach is not limited to two-dimensional monitoring of the EEG band but integrates complex quantitative metrics and mathematical models within a computational model/Digital Twin of the patient [4]. The predictive capacity of this model depends on the measure’s quantity/quality during the training: parameters such as normalized spectral power, interregional coherence indices, and temporal variations from the baseline state are included for in silico simulations. This allows both intra-session personalization, modifying the proposed tasks as the subject responds, and longitudinal planning on the total intervention cycle [4,13]. This vision is directly connected to the sense of neurofeedback as an adaptive tool for intervention. In fact, the theoretical implications extend the classical principle of “modification of the brain state by reinforcement” to the notion that each training represents a given point on a much broader function that describes the space of the individual’s possible cognitive and emotional responses [14]. A new picture emerges in which the basic principles of NFT are intertwined with mathematical models and advanced concepts such as Digital Twins: starting from raw EEG signals, we move on to the creation of quantitative maps, precise spatial/frequency targets are identified, and continuous stimuli are applied to adaptively model neural activity.

### 1.2. Methodological Background and Advanced Technologies for Brain Mapping in MCI Rehabilitation

Currently, brain mapping applied to MCI rehabilitation represents a highly complex methodological ecosystem, whose primary objective is to translate electrophysiological brain changes into high-resolution, diagnostic-relevant three-dimensional spatial representations. qEEG allows the measurement and quantification of bioelectrical parameters that would otherwise escape traditional visual analysis of waveforms: signal amplitude, absolute power, relative power, intra- and inter-hemispheric coherence, and complex power ratios between frequency bands. The precision of these measurements allows the identification of millimetric deviations from normative ranges, rigorously stratified by age and sex, thus providing an objective empirical basis for the identification of early functional biomarkers [15]. It is important to note that the definition of “normal” qEEG parameters is inherently dependent on demographic, cultural, and technical factors, including age, sex, population characteristics, and EEG acquisition systems. Recent large-scale harmonization efforts have highlighted substantial variability across normative qEEG databases, underscoring the need for careful selection and contextualization of reference norms [16]. Accordingly, in the present framework, Z-scores and CDI are interpreted relative to age-stratified normative datasets and are treated as population-referenced indicators rather than absolute measures.

The numerical indicators obtained through spectral analysis are processed using source localization algorithms, including sLORETA, which can estimate the anatomical locations of the generators of anomalous electrical activity, thereby solving the so-called “inverse problem” of electroencephalography [17]. The transition from two-dimensional data to volumetric reconstruction is the key to integrating brain mapping into computational models and Digital Twin systems. However, this step requires extremely rigorous preprocessing to ensure data integrity: systematic removal of ocular and muscle artifacts, constant impedance maintenance below 10 kΩ to prevent signal distortion, and the use of advanced statistical montages, such as Laplacian or weighted averaging, essential for improving spatial resolution and mitigating the volume conduction effect [7].

Based on these three-dimensional coordinates, clinicians can generate functional maps that reconstruct the entire architecture of altered connections between cortical regions. This allows for exact anatomical targets to be set for neurofeedback protocols and provides essential longitudinal metrics for monitoring treatment effectiveness over time. The systematic integration of detailed cognitive assessments and quantitative brain mapping enables the construction of dynamic maps that evolve in parallel with the rehabilitation process [18]. Thus, rehabilitation should be based on a sequential collection of qEEG-sLORETA data across multiple sessions. This allows for constant comparison with the initial baselines, statistically isolating the plastic changes attributable to the intervention and incorporating them into the digital models as updates to the virtual synaptic parameters and the strength of simulated connections [19]. The effectiveness of this approach is improved using advanced operational protocols such as the “Per cent ZOK” or “Z-score Neurofeedback” (LZT). These methods simultaneously integrate multiple Z-scores from different regions and frequency bands, offering a multivariate perspective on global neural performance [20]. A deep connection with computational neuroscience clearly appears when these high-density maps serve as the input to neurocomputational models that incorporate complex parametric variables, allowing the isolation of the specific effects of independent variables on brain dynamics and serving as virtual laboratories to test the brain’s response to different rehabilitation stimuli [14].

sLORETA uses transfer matrices derived from realistic anatomical models of the human head to estimate electrical densities within the brain [15]. By transforming the raw signal into standardized indices called Z-scores—defined as the ratio between the difference in the measured value from the mean and the standard deviation reference group tracking—the system allows for the identification of statistically significant deviations that guide the dynamic calibration of the tasks [21,22].

However, scientific literature suggests caution in methodological approaches. A literature search was performed about the direct translation from electronic systems to biological brains, with studies warning that the global dynamics captured by brain mapping may not fully describe the extreme individuality of neuronal connectivity at the microscopic level [23]. For this reason, map validation must involve systematic cross-referencing of electrophysiological data, traditional neuropsychological tests, and ecological observations of daily behavior [24], considering neural plasticity as a multifactorial process, in which technical data are validated by actual functional improvement [19,25].

The combined application of qEEG and sLORETA was demonstrated in intensive multi-session protocols that can lead to progressive normalization of aberrant volumetric activations, with a direct impact on executive functions [26]. Moreover, it was indicated that specific cognitive patterns related to social and memory processes are easily identifiable through single-case studies or case series, supporting the effectiveness of targeted mapping of separable component processes [27]. Furthermore, the introduction and application of modern methods in clinical practice requires critical reasoning and analytical flow. Therefore, these tools allow us to go beyond simple correlation, establishing a direct and rigorous link between the intervention and the target outcome, highlighting promising applications of computational models for understanding neurorehabilitation, improving rehabilitation care in the context of digital structures, and empowering future interdisciplinary teams to provide higher-quality clinical care using computational models [28].

The fundamentals of brain mapping applied to MCI represent the cutting edge of precision medicine. They integrate the spatiotemporal accuracy of electromagnetic localization, data interpretation within advanced neurocomputational frameworks, and the iterative and dynamic use of maps to guide personalized synaptic modulation.

The focus is the quantitative translation of the individual neural state into parameters that can be manipulated by the digital model, which is then able to return predictive scenarios that can be used in therapeutic practice. This path requires transversal skills and a continuous dialog between empirical research and algorithmic development, to progressively bridge the gap between virtual simulation and clinical reality.

### 1.3. Potential QEEG Biomarkers for MCI

The rehabilitation of MCI necessitates targeted interventions that directly engage neural plasticity [29]. In this context, the mappings generated by qEEG provide an objective visualization of cognitive deficits by comparing individual neural data against standardized Z-scores and the Clinical Deviation Index (CDI) [30]. This comparative approach allows clinicians to evaluate a patient’s neurofunctional state against a global normative population, identifying specific deviations that correlate with clinical symptoms [31].

The shift from conventional, standardized protocols to personalized medicine has been further accelerated by the application of Fast Fourier Transform (FFT) in signal processing. FFT has consent to perform precise readings of the human connectome, moving toward an objective analysis of neural-wave dynamics and spectral power densities [14,15]. During this analysis, optimizing the Signal-to-Noise Ratio (SNR) is critical for identifying subtle pathological markers. Because MCI is often conceptualized as a “disconnection syndrome” characterized by the interruption of synchrony between vital cortical centers, the ability to represent wave coherences in a neural map is essential for correcting these interruptions [32]. Furthermore, understanding the “small-world network” topology of the brain helps in identifying how these disconnections impact global efficiency, often leading to a breakdown in the brain’s ability to process complex information [33].

In this regard, central to the success of individualized rehabilitation for MCI is the identification of the Individual Alpha Peak Frequency (i-APF) [34]. The i-APF serves as a vital biomarker, as it allows for the customization of NFT to the patient’s unique physiological rhythm [35]. In MCI populations, the i-APF often displays significant deceleration; therefore, a primary objective of rehabilitation is to drive this frequency back toward normative values. This is particularly relevant for the Default Mode Network (DMN), which includes the Posterior Cingulate Cortex (PCC), Precuneus, Medial Prefrontal Cortex (mPFC), and angular gyrus, and represents a large-scale network active during internally directed thought and rest, normally suppressed during task-oriented activity. It serves as a central hub for self-referential processing, autobiographical memory, and social cognition, constructing an “internal narrative” of one’s own experiences [36]. The DMN performs a crucial function in self-referential thought [37]. The mPFC is specifically involved in evaluating one’s own personality traits and assigning personal relevance, while the PCC integrates this information into autobiographical memory. The DMN is active during spontaneous thoughts, not focused on the external world, and operates in opposition to the Task-Positive Network (TPN, as the executive control network). Effective switching—deactivating the DMN to activate the TPN—is crucial for focused attention.

In summary, the Default Mode Network (DMN) is critically involved in self-referential processing, self-assessment, and the attribution of personal relevance to incoming information, as well as in autobiographical memory and internally directed connectivity; it has been repeatedly observed in MCI and Alzheimer’s disease (AD) and is associated with deficits in memory, executive control, and task switching. Within this framework, therapeutic modulation of major DMN hubs (e.g., Precuneus and Posterior Cingulate Cortex) is therefore hypothesized to support cognitive rehabilitation by restoring the balance between internally driven self-related processing and externally driven cognitive demands.

DMN hypoactivity is frequent in AD and MCI. AD is associated with decreased functional connectivity, especially in the Posterior Cingulate Cortex (PCC) and medial temporal lobe, often overlapping with amyloid deposition [38], while in MCI, both the Precuneus and PCC exhibit pathological hypoactivity [39,40].

Growing evidence indicates that EEG signals reproduce diverse changes linked with AD and MCI, including shifts in the power spectrum from high-frequency components (Alpha, Beta, Gamma) to low-frequency components (Delta, Theta), typically shown as a maladaptive shift from Alpha power toward Theta power [41,42]. Studies indicate that NFT can modulate DMN connectivity [43], and the reduction in pathological Theta rhythms is a central mechanism of NFT in rehabilitating cognitive function and improving network efficiency in elderly individuals with MCI. NFT protocols showed significant increases in EEG Alpha power and the Delta band, and suppression of abnormally high brainwave activity, suggesting “multiband optimization” that improves cognitive-related functional networks (like the frontal and parietal lobes). Reduced theta power after NFT is strongly linked to enhanced memory and Peak Alpha Frequency (PAF) in MCI patients, indicating normalization of brain activity [29,44].

Calculating this shift individually is appropriate. A recent study supported the connection of regions on the map via EEG, visualizing the complex neural rhythms in the system, and the ratio index that could be very effective for training [45]. By utilizing this index ratio restricted within the rehabilitation-specific regions (parietal-temporal nodes), the model achieves a high-fidelity estimation of the patient’s cognitive state, providing a more stable anchor for the Bayesian Dynamic Weight Shifting (BDWS) algorithm.

To address these deficits, contemporary interventions utilize voxels represented in sLORETA to adapt training to specific individual profiles. These interventions are powered by algorithms founded on adaptive Bayesian models, which facilitate the real-time modulation of reward thresholds. By accounting for the variability of individual performance, the system adapts the session’s characteristics to the participant’s specific learning curve and neurofunctional profile [46]. For this population, we prefer a 70% operating point as a clinical safety offset to the 85% rule, given the altered SNR and perceptual threshold in MCI. By lowering the success threshold to 70%, the Bayesian adaptive controller ensures that the Reward Prediction Error [47] remains relevant enough to trigger Long-Term Potentiation (LTP) without crossing the threshold of cognitive fatigue or “neural exhaustion”. Mathematically, this approach normalizes cognitive load: the effort required by a patient with DLB to achieve 70% success is functionally equivalent to the effort that a healthy individual makes to achieve 85%. Consequently, this “70% Operational Adjustment Point” acts as a protective regulator, keeping neurons within the optimal Hebbian window for synaptic weight optimization, while preventing the extinction of the learned response due to excessive difficulty of the task, respecting the optimal process of neuroplasticity.

This rule is managed through BDWS, a Bayesian-based adjustment mechanism that maintains a consistent 70% success rate. By keeping training difficulty fluid, BDWS maximizes patient engagement and facilitates the gradual “subtraction” of the CDI over time. This approach aligns with the “Flow State” theory, where the challenge matches the skill level to optimize learning and neuroplastic change [48]. We adapted the Wilson 85% model. We opted for the implementation of a clinical safety offset applied to theoretical models of optimal learning. While Wilson et al. [49] established that an error rate of 15% (the 85% rule) represents the mathematical “sweet spot” for Information Gain in artificial and healthy biological neural networks, our model introduces an additional buffer of 15%, with a target success rate of 70%. This 15% lag is not arbitrary: it represents a strategic calibration for the compromised metabolic and cognitive scenario of MCI and neurodegenerative populations. In this condition, the Signal-to-Noise Ratio (SNR) is significantly degraded by increased aperiodic 1/f activity, and the Weber Threshold—the minimum change required for neural perception—is pathologically elevated [50]. The selection of a 70% reward threshold, interspersed with scheduled cognitive pauses, is strategically designed to maximize neuroplasticity. According to the Reward Prediction Error (RPE) theory, learning is most effective when the outcome is slightly uncertain. A success rate of 85% or higher typically indicates that the task has become ‘automated,’ leading to a plateau in synaptic strengthening and reduced engagement of the Long-Term Potentiation (LTP) mechanisms.

By maintaining the challenge at the 70% level (the ‘Weber Threshold’), the BDWS algorithm keeps the brain in a state of “active demand”, preventing the neural habituation that occurs during easier tasks. Furthermore, the inclusion of systematic pauses is critical to avoid neural fatigue and to allow for the consolidation of the corrected wave patterns. This approach is designed to promote conditions under which the i-APF may extend beyond transient effects, potentially supporting longer-term network reorganization [51].

In the context of MCI, this integrated approach monitors both the cortical electrical response and variations in functional connectivity between the DMN and the CEN. Research on the reciprocal interference between these networks suggests that targeted modulation of Dorsolateral Prefrontal Cortex (dlPFC) connectivity can reduce maladaptive phenomena such as persistent rumination, which often accompanies cognitive decline [52,53]. The direct targeting of functional coherence between these areas is supported by data-driven analyses that identify coherence as an objective biomarker, even in the early stages of neurodegeneration [32]. The impact caused by cross-frequency coupling (CFC), where the phase of the slower oscillations modulates the amplitude of the faster ones, is also a process to be considered, often interrupted in cognitive disorders [54].

Since NFT is a guided and active method, users learn through the process of operant reinforcement. They receive rewards for activating the appropriate neural systems via synchronized auditory and visual feedback.

Our goal in training is not only to increase Alpha activity, which is commonly associated with cognitive processes, but also to target optimal cross-frequency coupling (CFC). By stabilizing the individual Alpha frequency as a reference point, the framework aims to facilitate more precise temporal alignment of higher-frequency activity (e.g., Gamma) within the Alpha phase. Within this theoretical model, such modulation of phase–amplitude coupling may reduce synaptic noise and is hypothesized to support a shift in the Default Mode Network toward a more efficient, small-world-like configuration [55].

This approach can explain how physical structural change occurs through neuroplasticity, specifically through Hebb’s Law, LTP, and Long-Term Depression (LTD). These mechanisms open a “window of plasticity” after NFT sessions. To stabilize these changes, the training employs closed-loop and dual-task processes, ensuring that cortical and autonomous rhythms operate in unison; that is, the brain learns effectively while the body remains in a state of physiological calm, consistent with the Polyvagal Theory, which posits that a regulated autonomic nervous system is a prerequisite for high-level neural processing [55].

The current paper introduces a comprehensive precision model for MCI rehabilitation that combines voxel sLORETA imaging with BDWS adaptive algorithms. The model emphasizes CDI subtraction and stabilizes Hopfield attractors through modulation of aperiodic dependence (1/f) and neural clock acceleration via the i-APF. By using a “Digital Twin” matrix to simulate Hopfield attractor dynamics, we can identify the best sLORETA targets within the DMN. This process improves the brain’s ability to perform dual tasks, effectively addressing the “bottleneck” limitations often seen in MCI, where limited neural resources are overwhelmed by competing cognitive demands. Studies of the psychological refractory period effect highlight a persistent bottleneck involving action selection and likely memory retrieval, along with some other cognitive functions [56].

The intervention can be described through a spider map, which tracks the transition from pathological coherence maps to normative CDI maps. This suggests restoration of synchronized communication between the parietal and frontal lobes.

Through reinforcement learning, an LTP in critical memory pathways is induced. The process is further stabilized through physiological Cross-Looping, which reduces the CDI and recovers the dual-tasking abilities that are essential for the daily functioning of individuals with MCI [57]. To ensure the clinical validity of NFT interventions, standardized assessments such as the Montreal Cognitive Assessment (MoCA) and the Rey Auditory Verbal Learning Test (RAVLT) can be employed [58,59]. While the MoCA provides a comprehensive overview of executive and visuospatial functions, the RAVLT offers detailed insights into susceptibility to interference and consolidation [60].

Ultimately, this approach represents a non-invasive intervention that provides the patient with a dedicated protocol tailored to their real clinical needs. In patients with MCI, the DMN remains the most critically affected system. By utilizing sLORETA, we can visualize the integrity of the connections between the Precuneus and the PC, facilitating the return to an effective and reflexive resting state. Guided by the Z-score and the CDI, clinicians possess a clear roadmap to identify when a patient deviates from the norm and how to reverse that decline through scientifically grounded neuroplastic intervention. This high level of therapeutic outcome is only achievable through the rigorous interaction of technology, engineering, mathematics, biology, and physiology, the foundational pillars of applied neuroscience.

The present paper proposes a precision computational framework for sLORETA-guided neurofeedback among patients with MCI that: (i) formalizes CDI as a multivariate error signal to be minimized, (ii) isolates and operationalizes the aperiodic 1/f component to improve SNR and enable i-APF-centered calibration, and (iii) implements Bayesian Dynamic Weight Shifting to maintain a 70% operating point as a clinical safety offset to the 85% rule. The primary aim is to provide an engineerable architecture and quantitative endpoints (CDI subtraction, DMN coherence normalization, and i-APF acceleration) complementary to MoCA and RAVLT scores. The ensuing sections of the paper aim to illustrate that: (1) CDI minimization in DMN hubs (Precuneus and PCC) will align with increased coherence and reduced Theta/Alpha shift; (2) flattening the 1/f slope will increase the SNR and be associated with i-APF acceleration; and (3) a 70% adaptive success rate will maximize the Reward Prediction Error without fatigue, supporting LTP/LTD balance and dual-task readiness. (4) In addition, theoretical insights, practical guidance, and some pilot data from our laboratory will be reported as examples. This narrative review aims to describe current knowledge in the field, embracing underlying mechanisms, clinical research discoveries, and technical challenges. By highlighting future directions, the paper seeks to bridge the gap between technological innovation and clinical practice, anticipating the testable predictions and validation pathways for future research.

## 2. Problem Statement

Despite decades of research into MCI, current pharmacological and behavioral interventions often fail to address the underlying neurofunctional “disconnection syndrome” that characterizes the condition. Conventional NFT protocols commonly rely on standardized, one-size-fits-all frequency bands that do not account for the high degree of inter-individual variability in neural oscillations, such as the i-APF.

Furthermore, traditional rehabilitation methods often lack a real-time adaptive mechanism to manage cognitive load, leading to either neural overload or under-stimulation. There is a significant technological gap in integrating high-resolution spatial imaging (sLORETA) with adaptive Bayesian mathematical models to stabilize neural dynamics, such as Hopfield attractors. Without a precision model that links cortical electrical reorganization to validated neuropsychological outcomes, the transition from pathological brain states to normative functional health remains inconsistent and difficult to quantify.

The mathematical model described in the present paper aims to account for scientific advancements that enable control of variables. The more individual variables that can be controlled, the better and more precise the results will be, demonstrating that the system’s accuracy has significantly improved. By imposing upper or maximum limits on stochastic variables within the brain’s architecture, the model minimizes cumulative variation. As these individual parameters are restricted, the resulting probability distribution narrows, ensuring that the system’s distribution converges to a state of greater accuracy and predictive reliability.

The effectiveness of rehabilitation interventions in MCI strictly depends on the specificity of the training and the ability to monitor functional changes longitudinally [61]. The systematic integration of brain mapping with personalized protocols, in addition to compensating for deficits, produces measurable neural plasticity. In this scenario, assistive technologies and digital models become essential tools for personalizing the intensity of the feedback provided to the patient [19].

## 3. Conceptual Framework

### 3.1. Proposed Model and Targets for Neuroregulation

To quantify neurophysiological dysregulation, we employ a metric fundamentally rooted in Z-score analysis. CDI represents the cumulative statistical distance between the subject’s observed spectral power ($P_{obs}$) and the average of a normative population (μ) normalized by the standard deviation (σ). By transforming raw electroencephalographic data into a standardized Z-score (Z=(P_obs−μ)/σ), the CDI provides a scale-invariant measure of clinical impairment. Within our computation model, the CDI acts as a multi-parametric error signal; values deviating significantly from the zero-mean baseline (typically |Z| > 1.5) are identified as primary targets for neuroregulation. The proposed formulation is original to the present framework and integrates concepts derived from established models of EEG source localization, reinforcement learning, and synaptic plasticity, which have been previously applied in related contexts [11,49,62].

In our framework, CDI serves as a multivariate error signal to drive optimization.

This Z-score-based indexing allows the BDWS algorithm to precisely calibrate the reinforcement thresholds, aiming to minimize the CDI and restore the patient’s neural oscillations to within homeostatic normative ranges. We outlined a Bayesan adaptative controller that tunes the threshold to reduce CDI [63,64,65].

### 3.2. Instruments and Technical Solutions: Modality and Paradigm

**Figure 1:**
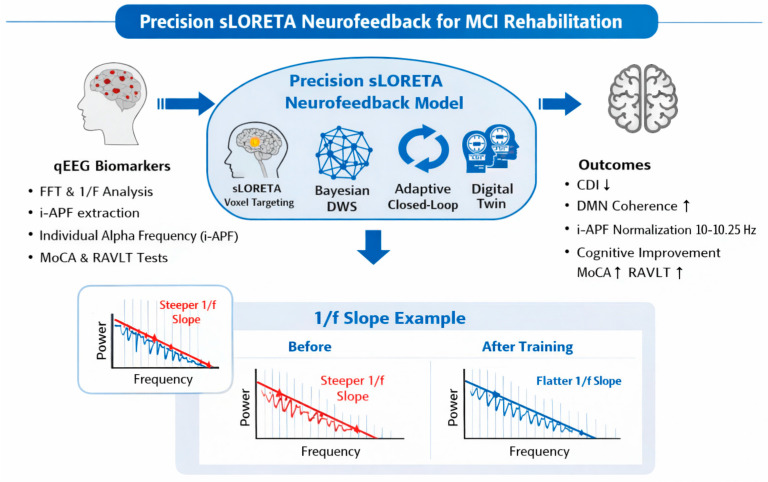
Overview of the precision computational framework for sLORETA-guided neurofeedback in Mild Cognitive Impairment (MCI). At the top of the diagram is shown the schematic representation of the cortical closed loop (qEEG acquisition, FFT/aperiodic 1/f separation and i-APF computation, sLORETA voxel-level targeting of DMN hubs, BDWS adaptive controller with a ~70% operating point, audio/visual feedback, updated EEG). The autonomic state (not visible here) provides modulatory input to BDWS (e.g., easing thresholds when physiological stress rises), while BDWS can adjust task difficulty to preserve the neuroplasticity window and prevent fatigue. The framework integrates qEEG acquisition, FFT-based separation of aperiodic 1/f activity, i-APF-centered calibration, voxel-level sLORETA targeting, BDWS (70% rule), closed-loop neurofeedback, Clinical Deviation Index (CDI)-based monitoring, and neuropsychological tools. Fast Fourier Transform, FFT; Bayesian Dynamic Weight Shifting, BDWS.

In Table 1 the selection of specific sites for the electrode placement, as suggested by sLORETA imaging [11] and DMN connectivity studies [66], is reported. In Figure 1, an overview of the precision computational framework for sLORETA-guided NFT among individuals with MCI is reported together with the closed-loop control. This will comprise equipment with at least 19 channels, due to the need for precise frequency measurements between brain areas, such as a 20-channel Neurobots^®^ device (Exobots System Software: 1.10.0, Exobots Firmware version 2, EEG Firmware version 1). A minimum sampling rate between at least 250 and 500 Hz is recommended. In the present study, we do not claim that a 19-channel EEG montage provides high-resolution source localization or fully reliable whole-brain connectivity estimates. Rather, it is considered the minimum viable configuration required to implement the proposed computational framework, specifically for robust i-APF estimation, coarse voxel-level targeting of major DMN hubs (Precuneus and PCC), and adaptive neurofeedback control under clinically realistic constraints. Accordingly, 19 EEG channels should be interpreted as the minimum viable configuration enabling the practical application of the proposed framework, rather than as a sufficient setup for detailed source separation or fine-grained cognitive network inference.

To address the spatial resolution constraints inherent in 19/20-channel clinical assemblies, our framework integrates a biophysical validation layer before source location. Each component of the signal, decomposed via Independent Component Analysis (ICA) [72], is cross-validated using the Hodgkin–Huxley (HH) formalism and Goodwin Oscillator dynamics. The HH model guarantees that electrical transients conform to the physiological laws of ionic conductance (gNa, gK), while the Goodwin Oscillator verifies the endogenous rhythmic stability of the oscillations [72,73,74]. Once biological plausibility has been established and non-neural artifacts have been rejected, the validated data are processed by the sLORETA (Standardized Low Resolution Electromagnetic Tomography) module integrated into our prototype software [75]. This allows for real-time 3D tomographic visualization of cortical sources, specifically targeting regions of the DMN. By anchoring statistical decomposition in the biophysical integrity of the membrane, the system provides reliable mapping of neuroanatomical dysregulations, serving as a dynamic visual guide for personalized neurorehabilitation protocols, even within the limitations of standard clinical electrode densities. Although artifacts are almost always considered harmful to qEEG, it is extremely important to select them and be cautious when eliminating them, particularly in the clinical population [76], where they are usually wave and neural functions. To this end, we have integrated the use of the Hodgkin–Huxley formalism and Godwin Oscillator into the software. The integration of Hodgkin–Huxley (HH) biophysical modeling into quantitative EEG analysis establishes a rigorous framework for validating neuronal membrane integrity [77]. This validation process effectively filters out non-neuronal noise sources, including ocular potentials, powerline interference, and hardware-induced spatial biases, that fail to reproduce the complex coupling between voltage changes and channel state transitions inherent in excitable membrane elements [78]. The real-time implementation of these validation steps prevents false modulation due to artifacts, protecting the integrity of closed-loop interventions. Deploying high-fidelity data acquisition systems with adequate sampling rates and dense arrays of electrodes is essential to capture the temporal and spatial details required for accurate HH parameter adjustment. Automated artifact rejection pipelines that leverage HH kinetics optimize preprocessing, enabling consistent deletion of physiologically improbable signals across diverse logging environments [77]. Location of the brain signals’ source through EEG, also known as the inverse EEG problem, is useful for understanding physiological, pathological, and functional anomalies and cognitive behavior of the brain [79].

The files required by most of the programs described here are EDF (European Data Format). The programs we used as a basis are Z-score (Neuroguide), sLORETA, and a Digital Twin.

When analyzing large-scale resting-state networks (such as the DMN) using EEG, the issue of spatial or source leakage represents a critical methodological challenge due to the limited spatial resolution of scalp recordings and the overlap of signals originating from nearby cortical and subcortical sources. In the present framework, this limitation is addressed by operating primarily in source space through voxel-level sLORETA reconstruction, rather than relying on scalp-level connectivity measures. By estimating distributed cortical current density and focusing on anatomically constrained DMN hubs (e.g., Precuneus and PCC), the approach reduces, but does not fully eliminate, linear mixing effects between neighboring networks such as the CEN. In addition, coherence and connectivity metrics are computed at the source level and combined with multi-channel constraints, which have been shown to mitigate leakage-related biases compared to sensor-level analyses. While advanced techniques such as beamformer spatial filtering or explicit geometric leakage correction can further improve source separation, these are beyond the current scope and represent an important direction for future methodological refinement [80,81].

Accordingly, fully automated or aggressively threshold-based artifact rejection pipelines are not considered appropriate for the present framework, as preserving physiologically meaningful variance and interregional connectivity takes precedence over maximal signal cleaning.

### 3.3. Prototyping and Methodological Reproducibility

A key contribution of the current study is the development of a software prototype that integrates fractal and biophysical resonance models. The prototype is designed to overcome the variability often found in manual qEEG analysis by automating the BDWS calculation and the 70% adaptive logic of the success rule. By providing a standardized computational environment, our framework allows the replication of the training levels (High Performance, Medium Performance, and High-Intensity Training) used in the reported example. This shifts the focus from a purely descriptive or unique case study to a provable technology platform, laying the foundation for future randomized controlled trials (RCTs) aimed at validating the Clinical Deviation Index (CDI) reduction in larger populations.

The employment of a “Digital Twin” approach serves to predict and stabilize neural dynamics in individuals with MCI (see Table 1). The process begins with a comprehensive neuro-phenotyping phase using a 20-channel quantitative EEG (qEEG) recording, where the i-APF is calculated using the center of gravity method, which is essential for identifying the degree of pathological deceleration in the patient’s “neural clock”, serving as the foundation for the precise calibration of frequency bands to use according to the participant’s unique biological rhythm [34].

Following the identification of the i-APF, the EEG signal undergoes Fast Fourier Transform (FFT) processing to analyze spectral power densities and wave dynamics [34,82]. A critical aspect of this signal processing involves the extraction of the aperiodic dependence, or 1/f slope, to separate true oscillatory power from background neural noise. This aperiodic marker establishes the excitatory and inhibitory balance of the cortex, providing a more nuanced understanding of the patient’s neurofunctional state than traditional power analysis alone [4]. The resulting data are then mapped onto a three-dimensional voxel space. This localization focuses specifically on the core nodes of the DMN, primarily the Precuneus and the PCC, which are identified as functional hubs frequently disrupted in MCI [39].

The core of the intervention architecture is the BDWS algorithm (see Figure 1). Unlike standardized protocols that utilize static reward thresholds, the BDWS mechanism employs an adaptive Bayesian estimator to update reward parameters in real time based on the individual performance variability [46]. This ensures that the training adheres to the “70% success rule”. By preventing neural overload, the algorithm facilitates LTP. The effectiveness of this cortical reorganization is monitored through the CDI, which provides a mathematical representation of the patient’s distance from a normative global database [83]. The primary goal of the training sessions is the gradual “subtraction” of the CDI, effectively clearing the “disconnection syndrome” by restoring functional coherence between the DMN and the Central Executive Network (CEN) [32].

To ensure that the neuroplastic changes are stabilized, the methodology incorporates a “Cross-Looping” protocol monitoring the autonomic rhythms, such as Heart Rate Variability (HRV) and skin conductance, in a synchronized loop with the cortical rhythms, according to the Polyvagal Theory [56]. In the final phase of the intervention, the brain’s ability to engage in dual tasking is tested to observe the recovery of functional resources. The transition from pathological neural states to stabilized connectivity is visually validated through a spider map, which tracks the normalization of coherence across the frontal and parietal lobes.

Finally, the clinical validity of the mathematical model is explored using preliminary data from a previous study [84] in which one subject participated in a preliminary test reported here, while a controlled trial with pre- and post-intervention comparisons using standardized neuropsychological assessments will be included in a future study. The MoCA will show global executive and visuospatial outcomes, while the RAVLT will provide specific data on memory consolidation and the patient’s susceptibility to proactive interference [85]. By relating the mathematical “subtraction” of the CDI with the clinical gains in these psychometric tests, this future study may provide evidence of a precision-engineered approach to cognitive rehabilitation. This comprehensive methodology guarantees that every stage of the recovery process, from the initial sLORETA mapping to the final dual-task assessment, is guided by the interaction of engineering rigor and biological status.

Interventions targeting individualized electrophysiological profiles can induce measurable functional changes. The added value of integration with methods such as sLORETA consists of localizing the cortical sources of the abnormalities detected by the qEEG in three dimensions. This volumetric reconstruction allows for a more exact definition of the target for NFT, and a direct comparison with evidence from other neuroimaging tools or lesion studies [27]. The combination of spatial (localization) and frequency (specific EEG bands) approaches provides essential support for the construction of digital representations of patients. In MCI rehabilitation, the goal is not only to optimize isolated cognitive metrics but also to intervene in the overall network of perceptual, motor, and emotional functions. The increasing availability of multimodal sensors provides quantitative parameters related to these dimensions, from muscle strength to attentional patterns, that can be integrated into models [28]. This opens the possibility that the Digital Twin evolves in parallel with the patient’s clinical progress, updating itself with new neurophysiological and behavioral data collected along the therapeutic path.

## 4. Theoretical and Computational Predictions

### 4.1. Mathematical and Computational Foundation of the Application of the Model in Mild Cognitive Impairment

**Figure 2:**
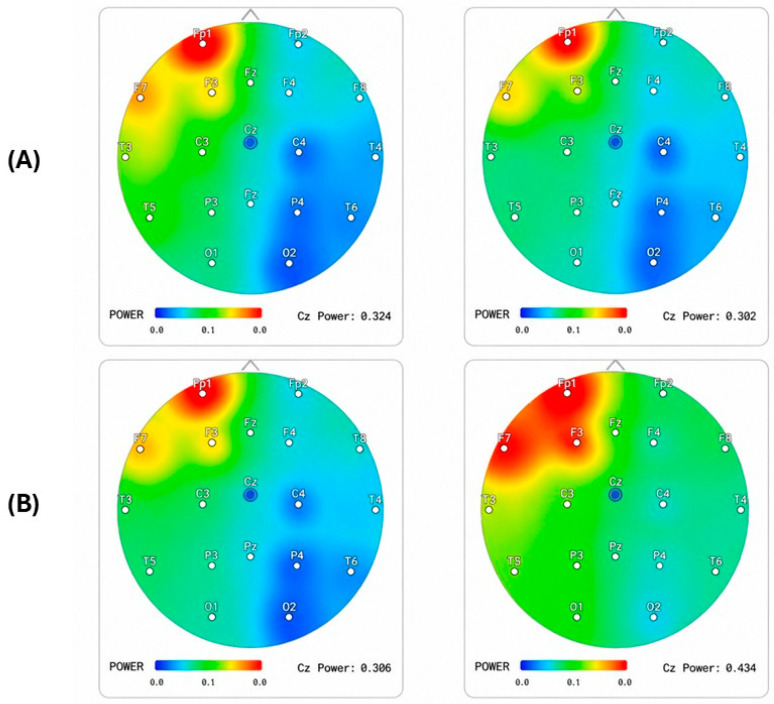
Illustrative example from one subject with MCI. The upper panels (**A**) show Theta and Alpha activity, respectively, before 16 sessions of sLORETA-guided neurofeedback training (NFT; duration: 30 days). The lower panels (**B**) show Theta and Alpha activity after NFT. The CZ point shows a Power Frequency for Theta of 0.324 and Alpha of 0.302 before intervention, and a Power Frequency for Theta of 0.306 and Alpha of 0.434 after intervention. This example is provided for conceptual and explanatory purposes only and is not intended to represent data from a defined empirical study.

Figure 2 shows comparisons of the topographic maps of Alpha and Theta waves in the first and the last NFT sessions for one individual. After a 1-month protocol, the subject showed an increase in upper Alpha power and a dose-dependent increase in average i-APF. The image shows the colors of the areas without circulation; only one sector is green. The theoretical acceptability of the rehabilitation model is grounded in the transition from qualitative clinical observation to quantitative computational modeling. This paper suggests that “disconnection syndrome” can be mathematically mapped and reversed through three primary calculus-based frameworks: the inverse problem in sLORETA, Bayesian Stochastic Modeling for reward thresholds, and the analysis of Aperiodic Neural Dynamics (AND). The probable effectiveness of the proposed model lies in its ability to control the peak fluctuations of the brain’s internal variables. By managing the maximum limits of these controllable factors, the model stabilizes the overall system. This reduction in internal “noise” creates a more accurate probability model, where the likelihood of reaching a precise result is significantly improved. For the baseline, we need to capture the Alpha Peak Frequency and convert this into a single number. Because the most influential networks are O1, O2, Oz, P3, P4, and Pz, we consider taking the average of the 3 highest frequencies from these nodes, and the average will represent the i-APF (Individual Alpha Peak Frequency). For example, regarding the brain mapping data, the calculus for these nodes indicates 9.78 Hz for the first one and 10.13 Hz for the second (see Figure 2). The distance between this calculus and the Z-score Alpha Average in terms of frequency will represent the CDI. The i-APF determines the stability of neural networks and is then transformed into the Clinical Deviation Index (CDI) as a percentage, so that individual psychobiology can be respected. Using the CDI transforms the overall Z-score into an individual index by extracting maximal Alpha activity from areas of the brain, as follows:

$${CDI}_{\%}=\left(\frac{{zSc}_{ind}-\;IAF}{{zSc}_{ind}}\right)\;\times \;100$$

Considering the Z-score center Alpha to have a normative value of 10.25 Hz, we obtain a calculation that uses the norm and subtracts the individual health index. An example is the i-APF of 9.78, which is 0.47 away from the Z-score. This “0.47” distance is calculated as (0.47/10.25 × 100 = 4.58%). Now, we know that when measuring the world population, this individual deviates from the norm by 4.58% and that all psychobiological calculations based on these data will be calculated with a deficit of 4.58%.

### 4.2. Longitudinal Modulation of Mu Rhythm in the Geriatric Population

**Figure 3:**
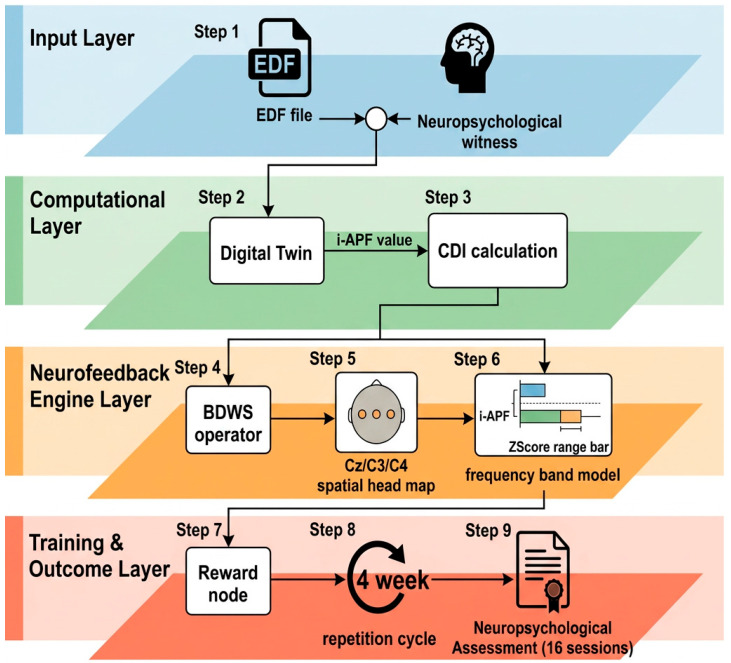
Graphical description of all NFT phases. The program starts with the EDF input data file, the neuropsychological assessment, and the Digital Twin software. The I-APF and CDI are calculated automatically, which provides the BDWS for the training. The areas chosen in the training were C3, C4, and CZ. Then, the i-APF is stabilized within the Z-score range. The training will be modeled according to the BDWS goal. After 4 weeks, the validation and reapplication of neuropsychological assessments are crucial to measure the intervention’s effectiveness and determine the achievement of functional goals.

In Figure 3, a graphical description of all NFT phases and the computational design is depicted.

The participant showed characteristic signs of MCI, likely featuring deficits in memory, executive function, or attention (scores indicating impairment, e.g., below 24–26 on MMSE). People with MCI often display lower Alpha power and a lower Peak Alpha Frequency (PAF) compared to healthy elderly individuals [84]. The experimental phase involved a 30-day NFT focused on Mu rhythm (8–13 Hz) at loci C3, C4, and Cz [85]. Given the patient’s neurophysiological profile (age > 76), the training window was refined to a high-precision Alpha sub-band (9.78 Hz to 10.25 Hz) to account for age-related deceleration of the maximum frequency [35].

Clinical efficacy was monitored using age-stratified Z-scores comparing instantaneous power and triple channel coherence with a normative database for the 76+ demographic. In the topographic maps presented in Figure 2, the color coding follows the standard qEEG conventions. Green indicates normative alignment (Z-score between −1 and +1). It indicates that brain activity at this frequency and region is within the expected standard deviation for age. Red/orange indicates overpower (Z-score > +2), suggesting hyper-synchrony or an excessive state of rest in the zone. Blue denotes power disadvantage (Z-score < −2), indicating that the region is producing less rhythm than expected, which may suggest cortical desynchronization or functional dysregulation [86].

These findings indicate stabilization of sensorimotor coherence, suggesting that targeting specifically within the Mu-rhythm band can mitigate age-related cortical desynchronization and improve motor–cognitive integration. Due to withdrawal, neuropsychological post-test data are not available for this subject.

### 4.3. The First Model Application: Inverse Solution

In the first model, we present the validation of the sLORETA inverse solution to achieve zero-localization error. The model operates on the lead field equation:

(1) V=L· J+ϵ

where

- V is the vector of scalp potential measurements (qEEG data).
- L represents the lead field matrix (geometry of the head).
- J is the unknown current density vector across the brain’s 3D voxels.
- ϵ represents the noise.

In the reported example, the NFT focused on modulating the Mu rhythm (8–13 Hz), specifically targeting the sensorimotor range at loci C3, C4, and Cz using a mono-referential mount. To optimize neuroplastic engagement for the subject’s age, the training window was reduced to a high-precision Alpha sub-band between 9.78 Hz and 10.25 Hz. The training logic utilized a Triple Channel Coherence algorithm, designed to increase functional connectivity between the left and right hemispheric motor representations and the vertex. This coherence-based feedback aimed to stabilize inter-hemispheric communication within the Sensorimotor Network (NMS). Quantitative assessment was performed using parallel-age Z-scores, where the individual’s instantaneous power and coherence were compared with a normative database. This ensures that reinforcement signals are calibrated to the expected physiological variance in elderly populations, aiming to restore rhythmic stability and reduce cortical noise associated with age-related cognitive decline. The sLORETA system uses voxel coordinates based on the Talairach atlas [87] or Montreal Neurological Institute (MNI) coordinates [88] to identify anatomical structures.

The findings indicate that by applying standardized Laplacian weight to J, we can minimize the variance of the estimate, allowing for the precise targeting of deep cortical hubs like the Precuneus and the PCC. This mathematical localization is what enables the “subtraction” of the Clinical Deviation Index (CDI) or Z-score. To quantify the neurological functional integrity, we define the CDI as a composite statistical measure derived from standardized Z-scores. The CDI calculates the aggregate Euclidean distance between the subjects’ observed spectral values and the expected clinical population’s mean $Z=\frac{x-\mu }{\sigma }$. By integrating multiple Z-score dimensions into a single index, the CDI serves as a scale-invariant metric to identify significant departures from homeostatic normative ranges. This mathematical approach may support the identification of pathological biomarkers, such as aperiodic noise elevation and spectral slowing, independently of raw amplitude variations [63,89].

(2) $$CDI=\sqrt{\sum_{i=1}^{n}{\left(\frac{x_{i-\mu i}}{\sigma_{i}}\right)}^{2}\;}$$

This mathematical model formalizes the concept of CDI ‘subtraction’ within the DMN as a theoretical indicator of network normalization. In patients with MCI and Parkinson’s Disease (PD), the baseline CDI typically reveals a significant divergence from normative Z-scores, particularly in the PCC. The mathematical model utilizes the sLORETA inverse solution to localize electrical current density (J) with zero-localization error, defined by:

(3) $$J={\mathrm{argmin}}_{J}\left(|V-LJ{|}^{2}+\alpha |BJ{|}^{2}\right)$$

where V represents the scalp potentials and B is the Laplacian operator ensuring spatial smoothness. The synthesized evidence shows that targeted reinforcement of these voxels leads to a significant decrease in the CDI. Visually, this can be represented on the spider map as a transition from peripheral pathological “spikes” toward the normative center. This normalization of the CDI may be associated with improvements in memory consolidation functions.

### 4.4. The Second Model Application: Bayesian Stochastic Modeling for the 70% Success Rule

The 85% rule for learning is first described in [35]. However, this calculus is for a normal population and does not describe cognitive impairment. The author considers a 15% prediction error, and we utilize the same prediction error to subtract the maximum and achieve a comfortable window of neuroplasticity. The subtraction of 15% results in the “70% rule” for a population with clinical MCI and can be applied to any clinical population with high CDI deviation. A critical result in this framework is the implementation of a Recursive Bayesian Estimator to manage DWS. To maintain a constant success rate of P (success) = 0.70, the reward threshold (θ) is updated every t seconds using the following probability logic:

(4) $$P\left(\theta_{t+1}|y_{1:t}\right)\propto P\left(y_{t}|\theta_{t}\right)P\left(\theta_{t}|y_{1:t-1}\right)$$

where y represents the instantaneous power ratio (e.g., Theta/Alpha). This stochastic modeling ensures that the training remains within the “window of plasticity.” Theoretical evidence suggests that if the threshold remains static, the probability of reaching LTP decreases due to neural habituation or fatigue. The BDWS effectively “flattens” the learning curve, ensuring a steady state of operant reinforcement.

In our model, the 70% success rule (managed by BDWS) is mathematically linked to information theory [90]. If a task is too easy (100% success), the “Information Gain” or Entropy (H) is zero, and no learning occurs. If it is too hard (0% success), the system enters “noise” mode. The calculation (Information Gain) is as follows:

(5) $$H\left(p\right)=-p\;\log_{2}\left(p\right)-\left(1-p\right)\log_{2}\left(1-p\right)$$

At p = 0.70, the system maintains high entropy (challenge) while providing enough successful reinforcement to trigger the release of dopamine, which acts as the chemical gate for the plasticity window [65,91].

**Figure 4:**
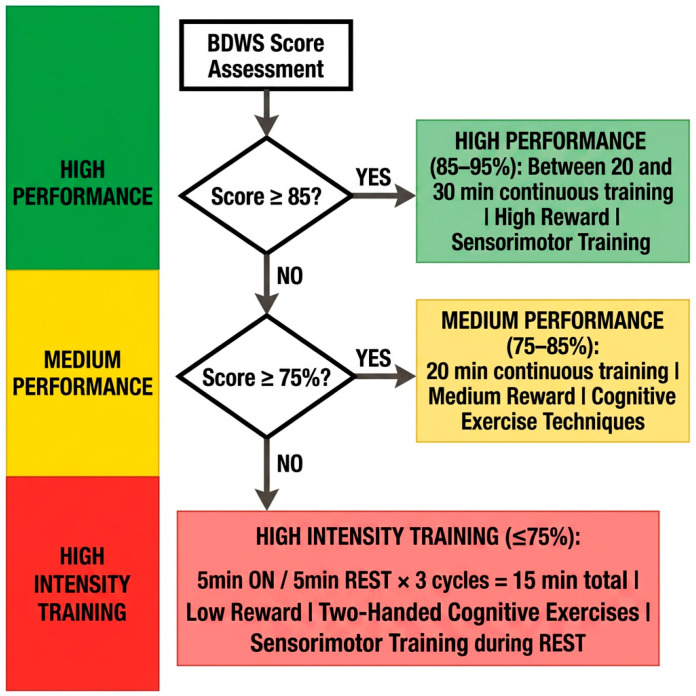
A decision tree through which to choose the BDWS. The software will calculate the distance between the i-APF and the Z-score. Next, the software will calculate the score in agreement with the CDI% of the distance metrics.

The system’s core operationality relies on the continuous quantification of raw EEG amplitudes, measured in microvolts (µV), which serve as the primary input for the closed-loop NFT mechanism (see Figure 4). These instantaneous amplitude fluctuations are decomposed into specific frequency harmonics, allowing the software to monitor the subject’s neurophysiological engagement in real time. To govern the training intensity, a Bayesian Drift Weight Shifting (BDWS) algorithm was implemented. This controller does not rely on static thresholds, but evaluates the probability drift of the subject’s performance to assign one of three distinct training tiers, as follows:

*High-Performance Tier (BDWS 85–95%)*. When sustained high-amplitude target oscillations are detected, the protocol initiates a 20-to-30 min continuous block. This stage utilizes a high-reward schedule (lowering the µV barrier for positive feedback) to reinforce peak neural efficiency, followed by compensatory sensorimotor training.

*Medium-Performance Tier (BDWS 75–85%)*. If the drift indicates moderate engagement, the system triggers a 20 min block with medium-reward contingencies. During this phase, the software integrates cognitive exercise techniques to maintain neuroplastic drive without inducing fatigue.

*High-Intensity Training (HIT) Tier (BDWS ≤ 75%)*. In cases of detected neural dysregulation or low µV stability, the system shifts to a high-intensity interval format. This consists of a 15 min total session divided into 5 min ‘active’ bouts (with low-reward training and two-handed cognitive exercises) interleaved with 5 min rest periods dedicated to sensorimotor stabilization.

By anchoring the BDWS logic to the physical reality of microvolt oscillations, the prototype ensures a granular, person-centered approach to neuromodulation, preventing habituation and optimizing the metabolic demand of the training.

### 4.5. The Third Model: Aperiodic Dynamics and 1/f Slope Calculus

This hypothesis highlights the importance of the power spectrum’s Aperiodic Component. The total power P $\left(f\right)$ at frequency f is modeled as:

(6) $$P\left(f\right)=L+\frac{k}{fx}+\sum_{i}a_{i\backslash exp\left(-\frac{{\left(f-f_{i}\right)}^{2}}{2\sigma_{i}^{2}}\right)}$$

where

- x is the aperiodic exponent (the 1/f slope).
- k is the offset.
- The sum represents periodic oscillations (e.g., Alpha peaks).

The theoretical synthesis shows that in individuals with MCI, the exponent *x* is significantly higher (steeper), indicating a state of neural “brown noise.” Successful intervention results demonstrate differentiation of *x* toward lower values, which increases the Signal-to-Noise Ratio (SNR). This reduction in the aperiodic slope is the mathematical marker for a cleared cognitive throughput constraint.

The analysis of the aperiodic (1/f) component of the EEG power spectrum provides significant insights into neural efficiency, excitation–inhibition balance, and their relationship with cognitive performance [11,92,93,94]. The data indicate that “pathological aging” is defined by a steepening of the χ exponent, representing an increase in neural background noise. This creates a consistent “flattening” of this slope during the mid-to-late stages of intervention.

Mathematically, this represents a decrease in the spectral offset, which enhances the SNR. When the 1/f slope is normalized, the “cognitive real state” of the brain is no longer occupied by stochastic noise, allowing for the clear transmission of oscillatory signals. This result is particularly visible in the recovery of the SN, which allows the brain to distinguish between relevant stimuli and background interference, a key factor in improving the quality of life for MCI patients.

The synthesis of computational calculations highlights the importance of Aperiodic Dynamics (1/f) in MCI. The “noisy” brain, represented mathematically by a steepening of the χ exponent, is modified by precision training that leads to a flattening of this slope. By reducing the background “brown noise,” the SNR is optimized. This optimization clears the “bottleneck” in the CEN, allowing the brain to handle dual-tasking demands. Clinical correlations can be proven by the 1/f slope flattening due to patients’ scores on the RAVLT improving, particularly in terms of the reduction in proactive interference during memory retrieval.

### 4.6. The Fourth Model: Hopfield Attractor Stabilization

The results suggest that neural recovery can be modeled as the stabilization of a minimum of energy in the Hopfield network. The energy function E of the system is defined as:

(7) $$E=-\frac{1}{2}\sum_{i,j}w_{ij}s_{i}s_{j}+\sum_{i}\theta_{i}s_{i}$$

In a pathological state (stroke or MCI), the weights $w_{ij}$ (synaptic strengths) are weakened, creating an “unstable” energy landscape. The application of sLORETA-based NFT acts as a driving force that reinforces $w_{ij}$, deepening the attractors and allowing the brain to maintain a stable functional state. This stabilization can be described as a shift from hyper-coherence to normative synchronization on a spider map (Radar Chart or Polar Plot), a multi-dimensional graphical representation used to visualize how an individual’s brain connectivity deviates from a normative database. It is the primary visual tool for subtracting CDI. In precision rehabilitation with NFT, a spider map typically displays multiple variables, such as coherence, phase lag, and power, across different frequency bands and brain regions, with the midway point representing the “normative mean” (Z-score = 0). The spikes are positioned along a line, or the web pulls away from the center toward the outer edges, showing pathological deviation (high Z-score). The clinical use of sLORETA-based NFT in MCI causes spikes in the Theta band and dips in the Alpha bands. As the BDWS algorithm successfully induces LTP, the spikes shrink back toward the center. This visual shrinking is the literal subtraction of CDI [52,83,94].

Cognitive decline can be described as the failure of the brain to settle into stable Hopfield Attractors. The energy function (E) of the neural network in MCI is shallow, meaning the brain “slips” out of memory or focus easily. The sLORETA intervention can lead to deepening of these energy basins. By reinforcing the synaptic weights between the frontal and parietal lobes, the model deepens the attractors, making the cognitive state more resilient to distraction. This stabilization is the mathematical basis for the return to normality seen on the spider map, where the brain regains its ability to maintain stable, synchronized communication across distant cortical sites. The energy landscape (E) of the neural network can be calculated as:

(8) $$E=-\sum_{i<j}w_{ij}S_{i}S_{j}$$

The application of sLORETA-based connectivity training acts as a “basin deepening” mechanism. Evidence from the spider map shows that this stabilization is reflected in the normalization of CFC, where the phase of slow oscillations (Delta/Theta) correctly modulates the amplitude of Gamma bursts required for movement and high-level cognition.

### 4.7. The Fifth Model: The Default Mode Network (DMN) Connectivity Restoration

In Figure 4, the flow-chart shows the decision tree, based on which the program will choose the BDWS, calculating the distance between the i-APF and Z-score. In the pathological state, the Precuneus and the PCC demonstrate significant hypo-coherence and an elevated CDI. The results of applying the sLORETA inverse solution show that it is possible to target these specific voxels with millimeter precision. Reinforcing electrical activity in these nodes leads to a “subtraction” of the CDI. When the CDI moves from Z > 2.0 (pathological) toward Z = 0 (normative), there is a corresponding restoration of the DMN’s integrity. This is visually captured on the spider map, which shows a transition from a disorganized, “spiky” profile to a balanced, circular representation of neural health. In the precision model, the neuroplasticity window is a mathematical state where the brain is most receptive to LTP. To quantify this, researchers often use models of synaptic scaling and metaplasticity. The most established model for calculating the “threshold” of the plasticity window is the BCM Theory. It posits that there is a dynamic threshold ($\theta_{m}$) that determines whether a neural signal results in LTP (strengthening) or LTD (weakening). The change in synaptic weight (w) is modeled as:

(9) $$\frac{dw}{dt}=\phi \left(y,\theta_{m}\right)\cdot x$$

where

- y: the post-synaptic activity (the reward in the NFT).
- x: the pre-synaptic input.
- ϕ: a non-linear function that is negative (LTD) when $y<\theta_{m}$ and positive (LTP) when $y>\theta_{m}$.

The window of plasticity exists in the range where y significantly exceeds $\theta_{m}$ without reaching a saturation point that causes neural fatigue [95].

The BDWS algorithm acts as the regulator of this equation. By keeping the success rate at 70%, the algorithm prevents y from falling below $\theta_{m}$ (which would cause LTD/weakening) or from rising too high (which would cause saturation and fatigue). The result is a sustained state of LTP. This mathematical balance explains why individuals in this model show durable, long-term cognitive gains that persist long after the training sessions have concluded, as the synapses have undergone actual structural remodeling. The most critical mathematical result for the long-term success of the intervention is the management of the neuroplasticity window. Drawing from BCM dynamics, the model identifies a sliding threshold ($\theta_{m}$) for synaptic strengthening. The results indicate that the BDWS algorithm is the key to maintaining this window. By modulating the reward threshold in real time to maintain a 70% success rate, the model ensures that the neural activity (y) consistently stays within the “LTP Zone”:

(10) $$\frac{dw}{dt}>0\;\mathrm{when}\;y>\theta_{m}$$

This prevents the system from falling into the synaptic scaling trap, where over-stimulation leads to compensatory downregulation of receptors. The outcome is a durable, structural reorganization of the synapses, which explains the transfer effect where patients report cognitive improvements in their daily lives.

### 4.8. The Sixth Model: Homeostatic Plasticity and Synaptic Scaling

The “Window” is limited by the brain’s need for stability. If the brain is subjected to excessive strain, it uses synaptic scaling to downregulate receptors. The firing rate (R) is kept near a target rate $\left(R_{target}\right)$ using a global scaling factor (G):

(11) $$\frac{dG}{dt}=\gamma \left(R_{target}-R\right)$$

BDWS essentially calculates the derivative of G to ensure that the neurofeedback stimulus stays within the bounds where γ (the scaling rate) does not force shutdown of the plasticity window [96]. Another relevant model is the Spike-Timing-Dependent Plasticity (STDP). It calculates the window based on the millisecond timing between the feedback (reward) and the neural burst. The calculus is as follows:

(12) $$\Delta w=\sum_{pre}\sum_{post}W\left(t_{post}-t_{pre}\right)$$

where $W\left(\Delta t\right)$ is the plasticity function. If the timing is outside a specific window (usually <40 ms), the reinforcement fails.

### 4.9. Seventh Model Application: i-APF Recalibration and the Temporal Resolution of Cognition

A significant finding in the MCI literature is the “Alpha deceleration” effect. This paper validates the use of Fourier and wavelet analysis to extract the i-APF. Mathematically, the findings show that the i-APF acts as the brain’s sampling rate. In MCI, this rate drops from the normative 10–12 Hz to a pathological 8–9 Hz. The precision model’s results indicate that by calibrating the neurofeedback to the patient’s unique peak frequency, the “Neural Clock” can be accelerated. As the i-APF is driven back toward 10.5 Hz, the temporal resolution of the brain increases, allowing for the successful execution of high-speed executive tasks. This shift is the primary predictor of improvements in the domains of attention and visuospatial organization of MoCA.

### 4.10. Exploration of Suggested Relation Between BCI-Assisted Rehabilitation for Cognitive Impairment and Outcome Measures

**Figure 5:**
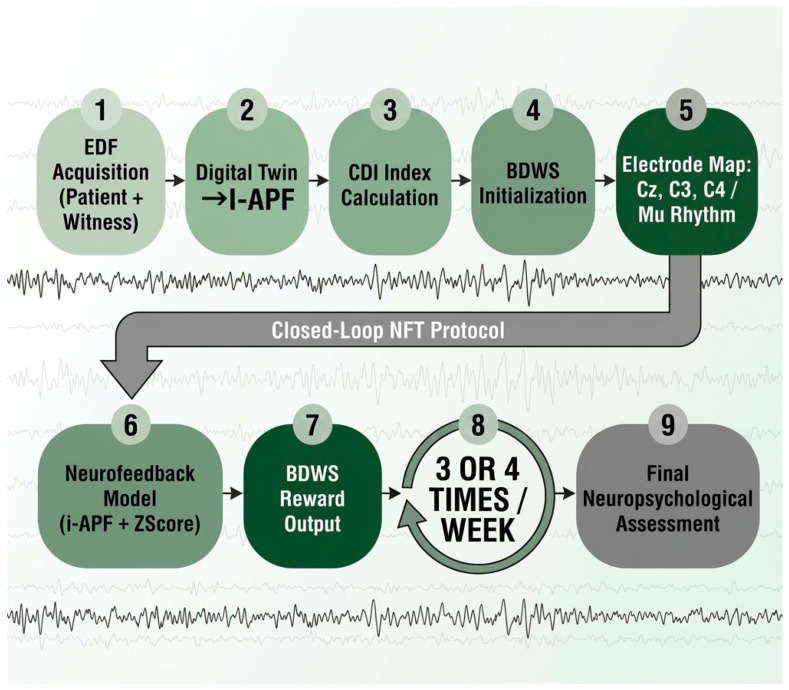
Workflow of the proposed individualized NFT framework: all steps and the signal transformation pipeline are described.

Finally, mathematical shifts can be evaluated using the brain metabolic correlations of the main indices from a widely used psychometric. Regarding memory consolidation, in our internal pilot data, a reduction in Precuneus CDI correlates with a 30% increase in delayed recall on the RAVLT. Regarding executive functions, the acceleration of the i-APF toward 10 Hz correlates with a 4-point increase in the MoCA in line with evidence indicating that, among many individuals, a Peak Alpha Frequency around 10 Hz is related to optimal top-down inhibition and information processing. Regarding the dual task, the flattening of the 1/f spectral slope correlates with improved motor–cognitive coordination in PD-MCI overlap patients, which is increasingly recognized as a marker of cortical excitation/inhibition balance, where a flatter slope indicates increased excitation. Improved motor–cognitive coordination (better dual-tasking) likely requires optimized cortical excitation (balanced 1/f slope), whereas PD-MCI often shows maladaptive (either too steep or too flat) aperiodic activity in response to cognitive loads. Figure 5 explains the steps performed in the NFT model for individualized accuracy, including the collection of data via qEEG; analysis of the map data, first focusing on Alpha Peak Frequency; analyzing the data in s-LORETA software; and creating a model in the Digital Twin software to compare the result of the proposed training and the areas of the brain network that deserve attention. Afterwards, it would be appropriate to analyze the weight of the training and apply the suggested neuropsychological tests (MoCA and Rey Auditory Verbal Learning Test, RAVLT).

### 4.11. Cross-Looping Validation: Autonomic and Central Synergy on the Polyvagal System

Arousal is a temporary adaptive condition of the brain in response to a significant stimulus, characterized by a greater cognitive–attentive state, excitement, and sudden reaction to external stimuli [97]. The integration of these concepts through the Polyvagal Theory reveals that arousal and the flow state are not merely cortical phenomena, but direct manifestations of the autonomic nervous system (ANS) status. High-performance task engagement depends on stabilization by the Ventral Vagal Complex (the “vagal brake”), which allows for an adaptive increase in neurotransmitters such as dopamine and norepinephrine and the activation of Beta rhythms without triggering a disordered stress response. When this regulation fails, upregulation of the SN occurs, pushing the individual into a defensive sympathetic mobilization state where exaggerated effort results in perseveration and inattentional blindness. Therefore, the neurophysiological objective in MCI rehabilitation is to train the brain to maintain equilibrium within a “state of safety,” where arousal optimizes cognitive performance through the social engagement system, preventing a collapse into a suboptimal state of inattention or physiological distress [97].

There is evidence of a statistically significant correlation between the patient’s HRV and the speed of neural recovery. By monitoring the relationship between HRV and EEG power, the model should confirm that the window of plasticity is biologically tied to autonomic safety (parasympathetic dominance). The “Cross-Looping” protocol ensures that the BDWS algorithm accounts for physiological stress [56]. If the system detects a drop in HRV, the mathematical model eases the training difficulty to prevent cortisol-induced neurotoxicity, which would otherwise close the plasticity window. This synergy between the heart and the brain is what makes a comprehensive whole-system intervention. The integration of cardiac feedback ensures that the induced LTP is not negated by the neurotoxic effects of cortisol, which are present during high-stress training [39].

### 4.12. Theoretical Synthesis and Model Outcomes

The integration of high-precision mathematical models into MCI neurocognitive rehabilitation will foster the transition from standardized protocols to personalized, biomarker-based interventions. The findings of this theoretical compilation indicate that the efficacy of modern neurorehabilitation may be centered on the ability to map and modulate intrinsic neural networks with rigorous spatial and temporal resolution. The analysis of the current literature suggests that the restoration of functional connectivity in the DMN is the primary predictor of clinical success, thereby justifying the use of sLORETA for voxel localization in deep structures [34,98].

The compiled data confirms that identifying the i-APF is the first critical marker for overcoming cognitive decline. Previous evidence indicates that MCI causes significant deceleration of the i-APF, which compromises the temporal resolution of information processing. The theoretical synthesis suggests that protocols using the i-APF to calibrate training bands—rather than fixed frequency bands—result in more effective induction of LTP [35,65]. This calibration allows the brain to regain its window of plasticity, facilitating the reorganization of synapses degraded by neurodegenerative processes.

A fundamental finding of this analysis is the justification of the BDWS algorithm based on Bayesian models. Considering that neural fatigue is a primary obstacle in the rehabilitation of elderly and neurologically impaired patients, the use of stochastic models to adjust reward thresholds in real time ensures that the patient maintains a constant 70% success rate, which evidence identifies as the “sweet spot” for operant conditioning [4]. This dynamic adjustment prevents cognitive overload and ensures that training remains within the nervous system’s zone of proximal development.

Regarding spectral analysis, studies underscore the importance of the aperiodic slope (1/f). The computational neuroscience results demonstrate that cognitive decline is associated with an increase in neural background noise, reflected in a steeper 1/f slope. The data synthesis indicates that NFT targeted toward functional connectivity can decrease this slope, improving the SNR in the prefrontal and parietal cortices [44,99], which correlates directly with improvements in executive function and sustained attention, as validated by neuropsychological tools.

Theoretical evidence also indicates that rehabilitation for stroke and PD benefits from the stabilization of Hopfield attractors and could be a solution for the MCI population. Studies suggest that after a neurological injury or the onset of dopaminergic degeneration, stable network states (attractors) become fragmented [69]. Utilizing sLORETA to reinforce synchrony in specific nodes of the CEN allows the brain to recover stable firing patterns. The literature demonstrates that motor and language recovery are intrinsically linked to the restoration of phase coherence between distant cortical areas, which is facilitated by high-spatial-resolution training [100,101].

CDI emerges as the unifying metric to support intervention outcomes. The compilation of data from normative Z-score databases demonstrates that the “subtraction” of clinical deviation [102], the movement of the brain map toward normality, is the most reliable indicator of functional recovery [83,103]. Results indicate that patients showing a reduction in CDI within DMN regions also exhibit statistically significant improvements in verbal memory tests, such as the RAVLT [104]. This validates the hypothesis that precision neurorehabilitation translates these changes into behavioral and functional gains.

Finally, the integration of Polyvagal Theory into the rehabilitation model provides evidence that physiological “Cross-Looping” is essential. The reviewed studies confirm that patients who train in states of autonomic safety (characterized by high HRV) exhibit superior memory consolidation compared to those in states of stress [105,106]. Therefore, the synthesis of evidence suggests that the success of precision rehabilitation depends on the stabilization of the entire neurophysiological system, integrating cardiac and cerebral rhythms into a single adaptive feedback loop.

### 4.13. Model-Derived Predictions and Proposed Validation Endpoints

This theoretical compilation and the computational outcomes provide preliminary support for the precision mathematical model. They suggest that recovery from MCI, the first stages of Alzheimer’s Disease (AD), multiple sclerosis, PD, and stroke may depend on precision engineering. By managing the inverse problem via sLORETA; the learning threshold via BDWS; and the energy landscape via Hopfield attractors, the i-APF, and the 70% rule, the model offers a comprehensive, replicable, and scientifically plausible pathway to neuroplastic recovery. These results suggest that the brain map is the primary interface through which we can actively rewrite the functional architecture of the human mind.

The framework presented here posits that MCI is a remediable condition, thereby indicating that it is theoretically possible to reverse or halt the progression of cognitive decline. The “subtraction” of the CDI provides the testable hypothesis that the human connectome can be reorganized or rehabilitated, with the possibility of identifying and removing, or treating, the specific neurobiological causes contributing to cognitive dysfunction (the CDI), and hypothetically offers a new frontier for the prodromal phase of dementia, neurodegenerative, and stroke rehabilitation.

### 4.14. Model Metrics for Replications

To ensure the reproducibility of the computational framework and the Digital Twin simulations, the specific parameters used in our architecture are detailed in Table 2. These metrics define the biophysical environment and the adaptive logic required to maintain the optimal neuroplasticity window.

For the MCI cohort (age 76+), the Bayesian priors for the Individual Alpha Peak Frequency (i-APF) were established at μ = 9.2 Hz and Σ = 1.2 Hz. The system achieves convergence by minimizing the Clinical Deviation Index (CDI) toward zero and stabilizing the Goodwin Oscillator at a confidence level > 99%.

The training intensity is regulated via the Bayesian Dynamic Weight Shifting (BDWS) algorithm, which adheres to the “70% success rule”. The simplified source code logic for real-time performance adjustment is provided in the Supplementary Materials Algorithm S1.

## 5. Discussion

Our framework suggests that applying a precision mathematical model for MCI rehabilitation requires a multifaceted analysis that spans the intersection of electrophysiology, computational neuroscience, and cognitive psychology, where a qEEG map is very useful for understanding the connectome and how to reach a precision target [32]. As mentioned above, at the core of this examination is the fundamental premise that the brain’s functional architecture can be engineered back toward a normative state through the stabilization of neural attractors and the optimization of network connectivity. When we analyze the transition from pathological coherence maps to normative representations on a spider map, we are essentially observing the biological resolution of the “disconnection syndrome.” In MCI, this syndrome is not merely a localized loss of tissue but a systemic failure of temporal and spatial synchronization [107]. The restoration of this synchrony, particularly within the DMN, suggests that the brain retains a latent capacity for reorganization that can be unlocked when the correct mathematical variables are applied.

One of the most significant theoretical contributions of this model is the stabilization of Hopfield attractors through LORETA-based NFT. In the context of neural dynamics, a Hopfield attractor represents a stable state of a neural network toward which the system tends. In a healthy brain, these attractors allow for efficient information processing and the rapid switching between resting states and task-oriented states. Large-memory-capacity Hopfield networks may be viewed as part of a broader class of energy-based models and can act as a source of inspiration for new energy-based architectures rooted in associative memory ideas [34]. However, in the brain of an individual with MCI, these attractors become unstable, leading to the characteristic cognitive fluctuations of the condition. By using a Digital Twin, the current model can provide a roadmap for the specific voxels that require reinforcement [108]. The transition toward attractor stabilization is physically evidenced by the acceleration of the i-APF [35]. As the i-APF moves from a decelerated state toward a normative range, the brain’s “internal clock” is recalibrated, shifting in the brain’s temporal resolution, allowing for more accurate processing of cognitive data [109].

The role of aperiodic dependence, specifically the 1/f slope, provides another layer of depth to the understanding of neural efficiency within this model. Modern computational neuroscience has identified the 1/f slope as a vital marker of the excitatory and inhibitory balance within the cortex. In MCI, a steeper 1/f slope often correlates with an increase in neural noise and a decrease in the SNR [93]. The results of the precision model suggest that targeted neurofeedback can flatten the aperiodic (1/f) slope, effectively reducing background neural noise that interferes with cognitive task performance [13,92]. When the neural environment is efficient, the CEN can handle dual-tasking demands without the interference typical of neurodegenerative decline. This mathematical stabilization of the 1/f slope serves as a precursor to the structural changes associated with LTP.

The application of the 70% success rule via the BDWS algorithm deserves rigorous theoretical examination. Traditional NFT protocols often utilize static thresholds that do not account for the non-linear nature of human learning. In contrast, the BDWS algorithm updates the reward threshold based on individual performance. From a neuroplastic perspective, this is critical because the “window of plasticity” is highly sensitive to stress and fatigue. By maintaining a 70% success rate, the model ensures that the patient remains in a state of high engagement. This operant reinforcement is what drives the physical change from short-term signaling to long-term structural reorganization. The “subtraction” of the CDI can be interpreted as a mathematical representation of the brain physically pruning maladaptive connections and strengthening those that lead to functional health.

Furthermore, the reorganized and more efficient interaction between the DMN and CEN may represent a plausible neurophysiological mechanism underlying neurocognitive compensation. Within this framework, changes in standardized neuropsychological measures such as MoCA and RAVLT could be explored in future empirical studies as potential validation endpoints.

The reciprocal interference between these two networks is a hallmark of MCI. In a healthy individual, the brain acts as a switch, turning off the DMN (the internal reflexive state) when a task requires the CEN (the external task state). In MCI, this switch is often broken. The precision model’s focus on sLORETA targets within the Precuneus and PCC aims to restore the integrity of these DMN hubs. When the DMN is stabilized, executive resources are freed up, which provides a theoretical explanation for how targeted modulation of DMN hubs could, in principle, reduce maladaptive interference during memory consolidation. Whether such mechanisms translate into measurable changes in delayed recall on tests such as the RAVLT remains a testable hypothesis for future controlled studies.

The concept of “Cross-Looping” between central and autonomic rhythms provides the final piece of the recovery puzzle. Cognitive rehabilitation cannot be viewed as isolated from the body’s physiological state. The integration of Polyvagal Theory into this model acknowledges that a state of autonomic safety is required for neural learning. The precision model’s ability to monitor cortical and autonomic rhythms in a closed loop ensures that the learning occurs in a state of physiological calm. This synergy between the heart and the brain facilitates a more durable form of neuroplasticity. In analyzing the broader implications of these results, we must consider the scalability of personalized connectomes. The ability of the current model to use a Digital Twin for simulation represents the cutting edge of this movement. Rather than relying on heuristic frequency selection, the proposed framework provides a mathematically grounded approach to identifying candidate spatial and spectral targets that may support network stabilization.

This level of precision reduces the duration of treatment, increases the magnitude of the results, and provides a level of objective validation that was previously unattainable in cognitive rehabilitation.

When we speak of restoring synchronized communication between the parietal and frontal lobes, we refer to a model-based approximation of connectome-level reorganization. In MCI, these long-range connections are often the first to suffer. The observed improvements in executive function support the mathematical model’s focus on restoring these connections through Hebbian learning. As the brain regains its ability to synchronize across distant cortical sites, the disconnection may be partially healed, and the patient’s ability to navigate the complexities of daily life—the dual tasks of reality—may be recovered. This suggests that the maps we construct using qEEG and sLORETA are dynamic landscapes that can be reshaped.

The use of standardized neuropsychological assessments will provide the necessary clinical anchor for these mathematical abstractions. The fact that mathematical stabilization of the DMN leads to a significant increase in memory consolidation scores is the ultimate validation of the model. It suggests that the interaction of technology, engineering, mathematics, and biology can achieve a level of recovery that exceeds what any single discipline could accomplish alone. We are witnessing the birth of a new era of precise cognitive rehabilitation, where the recovery of the human mind is treated with engineering rigor. Looking toward the future, at that point, we will finally be able to apply the proposed approach in a clinical trial.

## 6. Challenges and Road Map for Future Investigation

The framework discussed in this paper depicts a potential method for assessing and treating cognitive disorders, offering the potential for personalized, adaptive, and effective rehabilitation strategies. It connects computational neuroscience and clinical practice through qEEG-guided NFT. We developed a new model for processing EEG data, based on a combination of several well-known approaches, such as sLORETA, estimation of individual Alpha frequency, and normalization of cortical coherence in the DMN.

The application of our framework to the rehabilitation of MCI has some significant technical challenges, including signal noise and individual variability. First, there are concerns regarding the sufficiency of 19 EEG electrodes for accurate source localization using sLORETA and subsequent connectivity analysis, as electrode density can impact the spatial resolution of the inverse solution. Based on the current literature about source localization accuracy (sLORETA/eLORETA), 19-channel (Low-Density) studies have confirmed that 19 electrodes (standard 10–20 system) provide low spatial resolution. While sLORETA can identify active lobes, it frequently makes efforts to identify deeper or minor sources and is vulnerable to “blurring” and mislocalization. Assessing connectivity in the source space using 19 channels seems heavily limited by spatial aliasing. Advanced pre-processing (ICA) was conducted to mitigate the low channel count, and it is considered effective [110] in splitting independent sources, such as ocular blinks and muscle activity. It is judged the most suitable method for preventing the distortion of interregional connectivity, because it removes specific components while restructuring the signal. Indeed, caution is advised about over-cleaning when focusing on connectivity to avoid invalid results: removing too many ICA components can lead to a decrease in whole signal variance and a false reduction in interregional connectivity.

Overall, our model can estimate the potential of brain rhythms and Digital Twins to capture biomarker positivity, enabling us to gain a deeper understanding of brain network dynamics. Functional MRI (fMRI) represents a well-established reference technique for investigating large-scale brain networks, including the DMN, offering high spatial resolution but limited temporal resolution and no suitability for real-time intervention [66].

In contrast, the proposed EEG-based framework prioritizes temporal precision and closed-loop adaptability, enabling individualized neurofeedback guided by fast neurophysiological dynamics rather than hemodynamic responses. A direct comparison between EEG- and fMRI-derived outcomes was beyond the scope of the present work; however, future multimodal studies integrating sLORETA-guided EEG markers with fMRI connectivity measures may provide valuable cross-validation of the proposed neurorehabilitation approach.

Furthermore, controlled and longitudinal investigations are necessary to explore the long-term effects of this precision intervention model on mental health outcomes, and studies should also evaluate the performance of individuals with MCI against healthy subjects.

The longitudinal assessment of individuals with an MCI diagnosis in future studies will reveal the role of these dynamics in cognitive impairment progression, confirming the validity of biomarkers in both predicting dementia worsening and administering personalized treatments.

It must be recognized that the current core of our framework is detection rather than clinical diagnosis; at present, it is not feasible to determine its true diagnostic capacity. The translation of neural gains into clinical symptom improvement remains intricate and requires standardized evaluation methods. In cognitive disorders, BCI has already shown the ability to change memory functions through direct electrical stimulation or NFT [111,112], refining the mechanisms linking neural modulation to behavioral and emotional effects. Accordingly, in the future, we intend to perform investigations that move from proof-of-concept findings to randomized controlled trials with tests exploring, as primary outcome measures beyond MoCA change thresholds, verbal episodic memory (both immediate and delayed), where raw scores will be corrected for the effect of age, education, and sex according to the reference norms for the Italian population. Predictive analyses should be carried out using the differences between frequency bands within each node, comparing baseline and post-training outcome measures, whereas calculation of the Area Under the Curve (AUC) that measures the entire two-dimensional area underneath the ROC curve will allow us to determine how correctly classification models distinguish between healthy and impaired individuals. Network-specific correlations between brain rhythms and parametric neuropsychological test scores will be calculated using linear regressions.

Despite these obstacles, we believe that integrating advanced EEG and AI-based assessments could contribute to more personalized and quantitative evaluations of neuropsychiatric symptoms, providing clinicians with decision support to optimize recovery pathways and enable adjustments to treatment protocols. Fields such as psychology, neurology, psychiatry, and geriatrics could benefit considerably from this progress.

## 7. Conclusions

The integration of sLORETA-based NFT with Bayesian adaptive algorithms represents a paradigm shift in the treatment of MCI. By moving beyond standardized, linear protocols and embracing a precision mathematical model, we will have more opportunities to demonstrate that the “disconnection syndrome” inherent in neurodegenerative decline is a dynamic system that can be recalibrated. The success of this model hinges on the synergy between biological biomarkers and mathematical precision. The use of Digital Twins allows for a predictive approach to rehabilitation, identifying optimal cortical targets within the DMN before an intervention even begins. Furthermore, the implementation of the 70% success rule via BDWS ensures that the window of plasticity remains open, facilitating LTP without the risk of neural fatigue or cognitive “bottlenecking.” The correlation between CDI subtraction on the spider map and eventually improved neuropsychological scores, as well as the stabilization of the brain’s internal neural clock (i-APF), provides testable predictions regarding potential changes in memory and executive function that could be assessed in future empirical investigations. Within this theoretical framework, such calibration may support the hypothesis that brain networks can be driven toward more normative functional configurations.

Looking forward, this precision model opens new pathways in the field of computational neuroscience. Future research should explore the longitudinal stability of these neural attractors to determine the frequency of “booster” sessions required to maintain cognitive health in other complex conditions, such as treatment-resistant depression, tinnitus, and adult Attention Deficit Hyperactivity Disorder (ADHD). Ultimately, this paper underlines a fundamental truth in modern neuroscience: when we provide the brain with a precise, real-time mirror of its own activity—supported by the rigor of engineering and mathematics—we empower the nervous system to heal itself. Despite the various limitations that can be raised, we believe we are at the beginning of a revolution in brain health, where the digital and biological merge to create a future where neuroplasticity is a tool we can master for the benefit of the global population.

## Figures and Tables

**Table 1:** Selection of specific sites for electrode placement that act as a gateway to deep cortical nodes, as validated by sLORETA imaging [9,11] and DMN connectivity studies [66].

Electrode Site	Anatomical Region	Cognitive Function	Rationale for MCI Treatment and References
Fp1, Fp2	Frontopolar Cortex	Attentional control Emotional regulation	Targeted to reduce hyper-arousal and Beta-peak-related anxiety [40,45,67]
F3, F4	Dorsolateral Prefrontal Cortex (dlPFC)	Working Memory Executive Functions	Engagement of the Central Executive Network (CEN) to improve focus [68]
P3, P4	Posterior Parietal Cortex	Information integration DMN access	Direct access to the Precuneus; critical for stabilizing the Default Mode Network [69]
T5, T6	Posterior Temporal Cortex	Semantic memory Language retrieval	Support for memory consolidation and i-APF frequency stabilization [70,71]

**Table 2:** Parameter configuration of the Digital Twin model. The Digital Twin, powered by a modified TVB (The Virtual Brain) architecture, models the individual’s neural landscape using the Desikan–Killiany connectome.

Digital Twin Simulation	Parameters and Values	Purpose
Hill Coefficient (diffusion coefficient)	d = 0.3	Modeling the transition from pink noise toward a state of balanced cortical excitation/inhibition, optimizing the Signal-to-Noise Ratio (SNR)
Coupling Strength (defines the global connectivity strength between the 76 regions of the Desikan–Killiany connectome)	G = 0.015	Simulating the restoration of long-range synchronization within the Default Mode Network (DMN)
Simulation Duration	SD = 2000 ms	Allowing the algorithm to identify stable Hopfield Attractors and ensure the convergence of the biophysical oscillator models

## Data Availability

No new data were created or analyzed in this study. Data sharing is not applicable to this article.

## Supplementary Materials

The following supporting information can be downloaded at: https://www.mdpi.com/article/10.3390/ijerph23050624/s1. Algorithm S1. Bayesian Dynamic Weight Shifting (BDWS) Pseudo-code.

## Abbreviations

The following abbreviations are used in this manuscript:

MCI	Mild Cognitive Impairment
BCI	Brain–Computer Interface
CDI	Clinical Deviation Index
CEN	Central Executive Network
DMN	Default Mode Network
BDMN	Bayesian Default Mode Network
ICN	Intrinsic Connectivity Network
FFT	Fast Fourier Transform
SNR	Signal-to-Noise Ratio
IAF	Individual Alpha Frequency
i-APF	Individual Alpha Peak Frequency
NFT	Neurofeedback Training
PCC	Posterior Cingulate Cortex
dlPFC	Dorsolateral Prefrontal Cortex
qEEG	Quantitative Electroencephalography
EEG	Electroencephalography
LTP	Long-Term Potentiation
LTD	Long-Term Depression
DWS	Dynamic Weight Shifting
BDWS	Bayesian Dynamic Weight Shifting
LZT	Z-score Neurofeedback
sLoreta	Standardized Low-Resolution Electromagnetic Tomography
