# Supplementary material for: A Precision Computational Framework for sLORETA Neurofeedback in Mild Cognitive Impairment: Integration of qEEG Biomarkers and Neuropsychological Metrics
Source: Int J Environ Res Public Health. 2026 May 8;23(5):624. doi: 10.3390/ijerph23050624 (PMC13207330; doi:10.3390/ijerph23050624)
Supplement: Supplementary file 1 [file ijerph-23-00624-s001.zip › ijerph-4181466-supplementary.pdf]

Supplementary Materials

Supplementary Material S1. Bayesian Dynamic Weight Shifting (BDWS) Pseudo-code

The following pseudo-code describes the core logic of the Bayesian Dynamic Weight Shifting (BDWS) adaptive controller implemented in the proposed framework. The algorithm regulates training intensity based on real-time performance metrics and enforces a target success rate of 70% (clinical safety offset) to explore an optimal neuroplasticity window while mitigating the risk of neural fatigue.

Algorithm S1. BDWS Adaptive Controller Logic for MCI Rehabilitation

```
# BDWS Adaptive Controller Logic for MCI
Rehabilitation
# Target Success Rate: 70% (Clinical Safety Offset)

def update_training_tier(bdws_score,
performance_rate):
	# Performance_rate is the real-time reward hit
rate (Target = 0.70)

	# 1. Bayesian Threshold Adjustment
	if performance_rate > 0.70:
		decrease_reward_threshold ( )		#
Make it harder to maintain information gain
	else:
		increase_reward_threshold ( )		#
Make it easier to prevent learned
helplessness

	# 2. Training Tier Assignment based based
on Wellness Score
	if bdws_score >= 85:
		return "HIGH PERFORMANCE"	#
20-30 min continuous; High Reward
	elif bdws_score >= 75:
		return "MEDIUM PERFORMANCE" #
20 min continuous; Medium Reward
	else:
	# High Intensive Training (HIT)
to prevent 'Neural Exhaustion'
	return "HIGH INTENSIVE TRAINING (HIT)"
# 5min ON  /  5min REST cycles
```
